# Nursery Cares

**Published:** 1888-02-04

**Authors:** 


### THE DOCTOR'S PULPIT.

### NURSERY CARES.

It has often struck the doctor in the course of his daily duties that many mothers are unduly anxious about their children, and that their anxiety defeats its own ends. Let it be granted that young children in many cases have not a very firm hold of life; that the nurse is often a comparatively inexperienced young woman, and a superlatively careless one; that the best nursery milk is not seldom of uncertain quality; and that where there are from two or three to seven or eight children, all under twelve years of age, something will inevitably go wrong almost every day. Let all these things be granted, we must still affirm that some mothers are much more anxious than the daily occasion warrants, and that their anxiety is injurious both to themselves and their children.

The natural condition of healthily-born children is one of health, and the health tends to increase and become more stable with age until middle life is passed. That is a general fact worthy of sober consideration. But many mothers are in such a constant state of dread that one would think dangerous illness was the rule and health the exception. Let any healthy mother, whose husband is also healthy, look back upon her past life since her marriage, and ask herself how many really serious illnesses there have been in the nursery during that time. Of course we know there are mothers who do not dare to look back upon the history of the nursery lest the floodgates of sorrow should be opened—gates which once opened can hardly be closed again. But these are the exceptions. It still remains true that among healthy and fairly prosperous families health is the rule, and the want of it the exception.

Mothers are not always logical persons, and an appeal to their reason does not meet with so ready a response as an appeal to their sympathy. Nevertheless, reason has its decided uses, and it is a plant which well repays the most careful cultivation. If a mother with an anxious tendency could fix this simple generalisation in her mind, that *health is the rule,* what days and weeks of anxiety she might be spared! An illustration will make this clear. A certain number of railway accidents happen almost every year; but those accidents bear to the safe journeys the proportion of perhaps one in a thousand. How foolish a man would be who should worry himself into a fever every time he took a railway journey because there was one chance in a thousand that he might get his leg broken! Travelling under such circumstances would be intolerable. That kind of conduct is as wise and no wiser than his would be who should wear a thick frieze ulster at midsummer, lest all the tailors in the empire should die before Christmas. A reasonable amount of reasoning power and a certain measure of faith in the general providence of Almighty God, is what every mother should cultivate as a part of her highest duty, The Registrar-General's report shows that for every single life which is taken away during the year forty-nine are left; in other words, taking town with country, the average death-rate all the year round very seldom exceeds twenty in the thousand.

It need not be expected that children will get through their early infancy without troubles. When it is considered how various are the structures which constitute a complete baby, how wonderfully delicate many of those structures are, and what peculiar functions some of them have to perform, it is a standing miracle that any baby ever survives to reach the age of manhood at all. The stomach is often disordered by ill-selected and worse prepared food; but the physiologist who reflects how many organs contribute their share towards the digestion of even a baby's food is never surprised when he sees a coated tongue, and hears that Master Tommy, aged six months, has "done nothing but scream all night." How could he help screaming when the food was decomposing and worrying him with a griping colic? But though he should cry it does not follow that he must needs also die. It will take a good many colics to bring him to that pass.

Teething, to many children, is a very painful and distressing process, and it is a common experience that many troubles of a minor kind accompany it. A constant wearing and grinding pain will disorder the nervous system even of a grown-up person, and a disordered nervous system is a common cause of indigestion. Can it be wondered at that young children, when passing through such a formidable experience as the cutting of teeth, should have as accompaniments, sometimes a deranged stomach and liver, sometimes restlessness with loss of sleep, and sometimes even convulsions of an alarming character? But though these attacks are natural and to be expected in many cases, they are not inevitable, neither are they, as a rule, dangerous. Besides, children are not always teething. Even the most determined "tooth-cutter" will have intervals of freedom from his bugbear, intervals in which it behoves his too-anxious mother to try to be happy herself and to let other people be happy too.

Some mothers keep themselves and the whole household in a perpetual fret about ailments in their children no more serious than a slight cold. This is really too bad; and the other members of the family, especially the breadwinner, have in these cases just ground of complaint. A man has been known to declare that his wife was never happy except when she was thoroughly miserable. If there be any of this class among our readers we will ask them to permit a word of direct address. If you are a good mother you are most probably a religious woman. Do you carry your religion into the nursery and into the dining-room? Remember that Providence has quite as much care for babies as for sparrows, and that duty commands you to show as serene a temper to your tired husband, when he comes home, as you display to Mrs. Clappertongue when she calls for afternoon tea. Over-anxiety on behalf of children is not merely foolish—it is wicked.

Do we then advise a callous indifference as the fittest condition of mind for the average mother? Decidedly not! No one has a deeper sense of the necessity for constant and intelligent care in the management and rearing of children than the occupant of the "Doctor's Pulpit." It may be affirmed with sober truth that for the want of this many children grow up feeble and delicate who would otherwise be strong, and many others, to their parents' life-long grief, do not grow up at all. By all means let every mother make it her duty to understand thoroughly and scientifically all about the management of children. Let her study the subject fully and constantly, and carry out in practice all the best principles she has been able to master in her reading. But when she has done these things, then let her be content, and leave her children trustfully in the hands of Providence. Nature ordains that the majority shall survive the period of infancy and grow to riper age; and even if one or more should be taken back to Him who gave the life, is not that sometimes a thing to be thankful for in after years? Mothers lose a great deal both in comfort and strength when they shut out the care of Almighty God, and prefer to depend entirely on themselves and their own foresight. A mind overburdened with cares is not a capable mind, and will often fail in an emergency where a soul in repose and trust would succeed. So we come back again to the point from which we started, and affirm that many mothers are unduly anxious about their children, and that their anxiety very often defeats its own ends.

